# Youth supporting youth online: A content analysis of peer counselling at the online chat of @ease

**DOI:** 10.1016/j.invent.2026.100940

**Published:** 2026-04-01

**Authors:** Anouk Boonstra, Emmy Simons, Rianne M.C. Klaassen, Nina H. Grootendorst-van Mil, Nynke Boonstra, Remco F.P. de Winter, Therese A.M.J. van Amelsvoort, Sophie M.J. Leijdesdorff

**Affiliations:** a Mental Health and Neuroscience (MHeNS) Research Institute, Faculty of Health, Medicine and Life Sciences (FHML), Maastricht University, the Netherlands; b Child and Adolescence Psychopathology, Master Mental Health, Faculty of Health, Medicine and Life Sciences (FHML), Maastricht University, the Netherlands; c Department of Child and Adolescent Psychiatry, Levvel, Duivendrecht, the Netherlands; d Department of Psychiatry, Erasmus MC, University Medical Center, Rotterdam, the Netherlands; e Department of Healthcare, NHL Stenden University of Applied Sciences, Leeuwarden, the Netherlands; f KieN VIP Metal Health Care Services, Leeuwarden, the Netherlands; g Department of Psychiatry, UMCC Utrecht Brain Center, Utrecht, the Netherlands; h Mental Health Institute Rivierduinen, Leiden, the Netherlands; i Section Clinical Psychology, Vrije Universiteit Amsterdam, Amsterdam, the Netherlands

**Keywords:** Youth mental health, Care accessibility, Peer support, Digital mental health, Online chat

## Abstract

**Background:**

Accessible early intervention is crucial given the high prevalence of youth mental health problems. In addition to walk-in peer counselling for 12–25-year-olds, the @ease centres in the Netherlands offer an online chat service. This study aimed to contribute to the limited literature on online peer support by analysing links between topics, counselling techniques, and young people's responses, informing larger future analyses and practice.

**Methods:**

In 2023, a total of 2145 online chat sessions were held with 1160 individuals. A randomized subset was drawn for feasibility of this exploratory retrospective mixed methods study (*n* = 26). For the qualitative part, hybrid latent content analysis was performed using Atlas.ti 24 to infer topics, techniques, and response types. User responses were categorized as indicators of either good *(*e.g. *elaborative, reflective)* or poor client collaboration *(*e.g. *closed, dismissive)*. A logistic regression analysis in SPSS 27 was conducted to explore associations between counselling techniques and response types.

**Results:**

Most-discussed topics were social and occupational problems, problems related to parents, professional help and diagnoses, intimacy and sexuality, and suicidality. Of the 623 user responses within the 26 online chats, 423 (67.9%) reflected good client collaboration. Collaborative responses were positively associated with peer counsellors' empathising/affirming (*OR* = 4.13, 95%CI 1.53–11.16, *p* = 0.005) and negatively linked with their asking closed-ended questions (*OR* = 0.23, 95%CI 0.11–0.47, *p* < 0.001) (also when combined with empathising/affirming: *OR* = 0.25, 95%CI 0.12–0.54, *p* < 0.001), the combination of empathising/affirming with giving advice (*OR* = 0.17, 95%CI 0.07–0.46, *p* < 0.001), and giving advice only (*OR* = 0.27, 95%CI 0.12–0.61 *p* = 0.002).

**Conclusions:**

Actively listening through empathising/affirming, without directly asking a question or giving advice, helped young people in opening up when accessing online chat peer counselling. Future research could leverage large language models to enable extensive, topic-specific analyses, as the present sample size warrants carefulness in drawing conclusions, though complying with standards for qualitative research and sufficient counts for quantitative analysing. Gained insights can help optimize online peer support for youth such as that offered by @ease.

## Highlights

• In online peer counselling, young people talk about functioning, parents, sexuality, help and diagnoses, and suicidality
• Peer counsellors’ empathising/affirming was associated with young people opening up at an online chat service
• Peer counsellors’ advice-giving was associated with poor collaboration by youth accessing an online chat service
• Content analysis can help optimize online peer support for vulnerable youth, yet such research is scarce.

## 1 Introduction

As the vast majority of mental health problems develop by the age of 25, early intervention is crucial in preventing both emergence and worsening (Kessler et al., 2005; Solmi et al., 2022). Globally, the lack of nurture for young people is a danger to society and barriers to mental healthcare need to be removed (McGorry et al., 2024). Barriers include waiting lists, financial costs, a lack of a youth-focus, and an unclear pathway to mental healthcare (Leijdesdorff et al., 2021; MacDonald et al., 2018). In addition, standard services, and especially inpatient settings, can have serious iatrogenic effects on youth (Zuccala et al., 2025). Furthermore, many young people face a harmful discontinuation of care at the age of 18 years instead of a well-organized transition to adult services (Boonstra et al., 2024a; Signorini et al., 2018). To ensure accessible, youth-tailored, and ongoing support instead, initiatives have been developed across the globe that provide soft-entry tailored care for young people aged around 12 to 25 years (Hetrick et al., 2017; McGorry et al., 2022).

Beside face-to-face interventions, progressively more accessible innovative digital mental health interventions have emerged for young people around the vulnerable age range of 12 to 25 years. Examples include Enhanced Moderated Online Social Therapy (MOST+) delivered through digital modalities (Alvarez-Jimenez et al., 2021, Alvarez-Jimenez et al., 2025), including the Dutch adaptation termed ENYOY (“Engage Young People Early”) (van Doorn et al., 2023), as well as an Ecological Momentary Intervention (EMI) called ‘SELFIE’, where exercises are tailored in real-time through an application in which self-esteem is targeted in youth who experienced childhood adversity (Reininghaus et al., 2024). However, digital peer support for young people with mental vulnerabilities has still scarcely been evaluated, especially in online chat environments (or: “synchronous written conversations”). Many examples of active online chat peer support services exist throughout Europe (e.g. PeerChat in the UK; Rat Auf Draht in Austria) but, to our knowledge, none have published scientific evaluations.

While less-researched among youth, the feasibility and acceptability of peer support through online chats has been shown among adults with e.g. lived experience of mental disorders (Fortuna et al., 2020), perinatal depression/anxiety (Baumel and Schueller, 2016) or schizophrenia-spectrum disorders (Baumel et al., 2016). In youth, other modalities of peer support were tested, for example one pilot study in the UK demonstrated feasibility and effectiveness of an online community peer forum platform (Stevens et al., 2022). In a systematic review on online peer support for young people aged 12 to 25 years, it was noted that studies usually include peer support as an add-on, instead of at the centre of support, and that the only found studies that exclusively tested peer support in online chats did so for smoking cessation (Ali et al., 2015). One study in the Netherlands analysed social support in peer-delivered online chat sessions in 2008 among 16–23-year-olds by use of a content analysis (Fukkink, 2011). More recent studies are limited, and research is needed to evaluate and improve practice, to help address the existing knowledge gap regarding online chat peer support for young people.

In the Netherlands, the @ease youth mental health foundation (www.ease.nl) was established in 2017, with its first peer support walk-in centre opening in January 2018. @ease is part of the global youth mental health movement for accessible and early intervention based on headspace Australia (McGorry and Mei, 2018; McGorry et al., 2022, McGorry et al., 2024). Such support is needed as over half of young people in the Netherlands experience mental health problems before the age of 24 years (ten Have et al., 2022). @ease is directed toward youth aged 12–25 years, designed through youth involvement, without waiting list, anonymous, and cost-free (Boonstra et al., 2023; Leijdesdorff et al., 2022). Accessibility is furthered due to all support being provided by a duo of trained and supervised peers, often with lived experience (Leijdesdorff et al., 2022). Significant improvements were found over @ease visits in young people's psychological distress and social and occupational functioning, and counselling satisfaction was high (Boonstra et al., 2024b). To evaluate and improve the service continuously, cost-effectiveness and follow-up effectiveness analyses are ongoing. Next to the primary walk-in service, @ease offers an online chat service that was launched in response to the challenges posed by COVID-19 restrictions in 2020, but the online chat service remained well-visited. However, no evaluation has been performed on the peer-delivered online chat sessions at @ease.

Anonymous online chat transcripts offer unique naturalistic insights in the arising topics, used counselling techniques, and chat-user responses. The aim of this study was to conduct an initial exploration of these three aspects and their potential interconnections, providing a basis for future in-depth analyses. First, topics were hypothesized to align with topics reported in the questionnaires at the end of walk-in visits. Young people have self-reported that the main reason for visiting @ease was to talk about ‘my feelings’, ‘social relationships’, and ‘education/work’ (Boonstra et al., 2024b). Less-chosen topics were ‘drugs/alcohol’, ‘physical health’, and ‘sexuality’, which may arise more readily in an online chat environment due to enhanced anonymity.

Second, peer counselling techniques were expected to originate from the @ease training. The training equips prospective peer counsellors with practice in active listening skills. Active listening, as coined by Rogers and Farson (1987), means to actively try to understand the other person from their point of view by hearing the meaning and feelings behind what is being shared, while avoiding judgment or trying to change the person's view or approach through convincing or advice-giving. Connected parts of the @ease training were solution-focused techniques and motivational interviewing strategies, as well as preparation for uncommon situations of crises. In an online setting, these trained elements may involve asking questions, empathising, providing appropriate self-disclosure, checking one's understanding, and stimulating thinking of solutions, instead of giving advice or proposing solutions (Leijdesdorff et al., 2022). Hence, we hypothesized that peer counselling techniques in the @ease online chat would primarily consist of asking open-ended questions, checking one's understanding, expressing empathy, providing self-disclosure, and stimulating a solution-focus. Conversely, we expected minimal use of giving advice and asking closed-ended questions.

Third, differences were expected in the response types based on the used counselling techniques. The degree of moment-to-moment engagement has been described in previous therapy research as high or low “client collaboration”, which has been linked with the effectiveness of counselling techniques (An et al., 2025). While young people visiting @ease are not “clients”, responses in the online chat were also best categorized as either good or poor collaborative responses, where collaborative means “being engaged” rather than “being cooperative”. Based on the peer training, we hypothesized that collaborative responses would be positively linked with active listening and related strategies and negatively linked with giving advice and asking closed-ended questions.

## 2 Methods

### 2.1 Design and procedure

When entering the online chat, young people are asked to enter a nickname, gender, age group, and a smiley to inform the peer counsellors about their mood. Next, the familiarity with @ease and the working method is enquired. Following this, the peer counsellors apply the @ease working method as described above to guide active listening. These aspects were part of the training delivered to the peer counsellors, which also included suicidality prevention training on how to talk about suicidal ideations and plans and when to involve the on-site professional who can contact an on-call psychiatrist if needed. This mental health professional supervises the conversations and is available for guidance and aftercare when needed (Boonstra et al., 2024b). Young people could find @ease in several ways, as it is promoted through social media, schools, community outreach, word-of-mouth, mental health professionals, and the website.

For this first study, online chat sessions were drawn one at a time from all transcripts between January and December 2023 using a random number generator, and each transcript was coded iteratively. Transcripts were drawn and coded sequentially until no new main themes emerged, based on redundancy and consistency. This approach combined the representativeness of random sampling with the qualitative principle of saturation as a stopping rule, allowing for identification of patterns across the dataset. Online chat sessions were excluded if they contained no response by the young person or were decidedly fictitious. If an online chat session was terminated (e.g. due to Wi-Fi connectivity issues), the separate segments of the conversation were matched together when the young person returned that day using an anonymous id-code generated by the chat provider. No decoding document was accessible to the researchers. The qualitative part of this study was reported by use of the COnsolidated criteria for REporting Qualitative research (COREQ) Checklist (Tong et al., 2007), detailed in Supplementary Table 1.

The Medical Ethical Committee of Maastricht University approved the @ease online chat study proposal and declared the study non-subjected to the Medical Research Involving Human Subjects Act (METC number 2023–0162). Transcripts were shared securely with the active researchers by anonymous online chat provider Newtee, who made potential identifying information unavailable to the researchers before the start of this study, filtering out e.g. locations or names of organisations that might have been mentioned in the transcripts. Documented consent was not required because no identifying information was processed and no information can be traced back from this article. Moreover, having to answer requested forms can generate a substantial obstacle for receiving quick and accessible peer support which is disproportional to the aggregated data where no identifying information is pre-processed and no personal stories published. Moreover, it would seriously hamper the ecological validity of the study while addressing this research gap is in the public interest. Evidence on how support is exchanged, and which factors contribute to helpful interactions, directly informs improvement of peer support, which is increasingly used as an accessible form of support given the mental health crisis in youth.

### 2.2 Participants

In 2023, a total of 2145 @ease online chat sessions were held, with 1160 individuals that had not accessed the online chat before. On average, 179 online chat sessions were held per month, including on average 97 online chat sessions per month with new visitors. As shown in Table 1, most participants were female (77.0% overall; 84.6% of the subsample analysed in the present study) and the majority was of the age groups 12 to 15 years (31.8% overall; 42.3% of the subsample) and 18 to 25 years (39.9% overall, 42.3% of the subsample). The subsample that was sampled through random number generation until no new themes arose, included 23 Dutch (88.5%) and three English (11.5%) conversations.

**Table 1:** Demographic characteristics of the participants in the online chat sessions^a^.

	2023 all (*N* = 1160)	2023 subsample for the present study (*n* = 26)
Age group^b^		
Younger than 12 years	16 (1.4%)	0
12 to 15 years	369 (31.8%)	11 (42.3%)
16 to 18 years	255 (22.0%)	4 (15.4%)
19 to 25 years	463 (39.9%)	11 (42.3%)
Older than 25 years	57 (4.9%)	0
Gender		
Male	189 (16.1%)	4 (15.4%)
Female	902 (77.0%)	22 (84.6%)
Other	22 (1.9%)	0

a Not all respondents chose to fill out these items.

b @ease is intended for ages 12–25 but others sometimes visit the chat as well.

### 2.3 Analyses

Qualitative analyses were performed in Atlas.ti 24 and quantitative analyses were performed in IBM SPSS Statistics for Windows, version 27. In content analysis (i.e. the analysis of textual data), a combination of qualitative and quantitative analyses is often required, where data must first be interpreted to then enable frequency-based analyses (Graneheim et al., 2017). Interpreting the texts was done through a combination of inductive (text-driven, from the online chat sessions' contents) and deductive (concept-driven, from hypotheses) approaches. The combination of latent content analysis and hybrid coding integrates existing knowledge with concepts found in the text to infer meaning (Bengtsson, 2016; Kleinheksel et al., 2020). A dataset between one and 30 transcripts is typical for qualitative research but sample size must be established based on informational requirements to answer the research question adequately (Bengtsson, 2016).

Online chat sessions were read and re-read to familiarize deeply with the data, before and throughout latent hybrid coding of both hypothesized and new topics, counselling techniques, and responses, by authors Drs. ES (she/her, psychologist, MSc Child and Adolescence Psychopathology) and Drs. AB (she/her, psychologist, final-year Youth Mental Health PhD Candidate), who had no connection to each other prior to this study. Five online chat sessions were first independently coded and discussed among ES and AB to brainstorm about the coding to establish the coding framework and ensure consistency. Subsequently, the remaining online chat sessions were coded by ES, with coding decisions, ambiguities, and any refinements being discussed collaboratively within the study team to maintain analytic rigor and reflexivity. Topics of high resemblance were merged, and minor topics were grouped into key topics. Sampling was done consecutively until no new themes emerged.

Next, descriptive analyses were conducted to summarize online chat session information. Correlations between the independent variables as well as between the independent variables and the outcome variable were computed to assess potential multicollinearity and to facilitate interpretation of the main analysis. Furthermore, while the total of 631 data points stem from 26 online chats and the data thus has a hierarchical structure, the limited sample size and low intraclass correlation of 0.002 did not support the use of a complex mixed-effects model. Therefore, a binary logistic regression model was constructed to explore which counselling techniques were linked with the response types. To correct for multiple testing (9 independent variables), a Bonferroni correction was applied, setting the significance threshold at *a* < 0.006.

## 3 Results

### 3.1 Characteristics

Of the 26 young people of whom the online chat sessions were randomly sampled for this study, 17 (65.4%) reported no prior familiarity with @ease, while nine participants (34.6%) were familiar with the service. Nine (34.6%) participants terminated the online conversation abruptly for unknown reasons. The chat duration spanned from four to 88 min, with an average online chat session duration of 39 min.

### 3.2 Online chat topics and counselling techniques

Throughout the 631 exchanges in the 26 online chat sessions, the researchers identified five overarching topics that led young people to seek online peer support at @ease. These topics were social and occupational problems, problems related to parents, intimacy and sexuality, professional help and diagnoses and suicidality, as detailed in Table 2.

**Table 2:** Frequency and distribution of topics in the 631 exchanges within the 26 online chat sessions.

	Number of times topics were mentioned throughout the 631 exchanges in the 26 chats	Number of online chat sessions in which topics were mentioned ⁎
Social and occupational problems a	307	21
Problems related to parents b	106	7
Intimacy and sexuality c	93	8
Professional help and diagnoses d	66	7
Suicidality e	59	7

⁎ Multiple topics could arise within an online chat session.

a Friendship, school, work, loneliness, inactivity due to somatic complaints, social difficulties.

b Bond with caregivers, domestic violence toward young person, parental alcoholic problems, parental divorce.

c Contraceptives, being in love, intercourse, masturbation, relationship acceptance by parents.

d Depression, trauma, panic attacks, professional help.

e Suicidal thoughts, police involvement regarding suicide, past help for suicidal thoughts.

### 3.3 Counselling techniques

Next, 76 distinct counselling techniques were found which were merged into 11 main categories, described in the codebook in Supplementary Table 2, with frequencies shown in Table 3. Peer counsellors listened to young people who sought digital peer support primarily by asking open-ended and closed-ended questions. Open-ended questions served to encourage the young person to express themselves and guide active listening (e.g. ‘*How are you feeling right now?*’), while closed-ended questions could induce a yes/no response or evoke elaboration (e.g. ‘*Did you enjoy that experience*?’). In that same line, peer counsellors also asked questions specifically to check their understanding, such as repeating a segment and ending with ‘*Is that correct?*’

**Table 3:** Number of times that counselling techniques were used (601 times in total) within the five main topics during the online chat sessions (*n* = 26).

	Problems related to parents	Professional help and diagnoses	Intimacy and sexuality	Social and occupational problems	Suicidality	Total
Asking a closed-ended question	24	5	8	61	15	113
Asking an open-ended question	22	5	21	45	17	110
Empathising/affirming	13	14	21	46	3	97
Checking understanding	4	7	0	6	3	20
Empathising/affirming and closed-ended question	13	10	6	37	2	68
Empathising/affirming and open-ended question	8	6	8	22	2	46
Giving advice only	10	5	11	27	5	58
Structuring the conversation	4	3	3	17	4	31
Empathising/affirming and giving advice	4	5	3	15	3	30
Stimulating solution-focused thinking	1	4	2	9	0	16
Providing self-disclosure/information	1	0	3	6	2	12
Total	104	64	86	291	56	601

*Note:* More mentioning of these topics was identified than could be linked to counselling techniques, hence, the columns specifying “total” are lower than in Table 2.

Another used counselling technique was to structure the conversation, for example: ‘*You name several things. I would like to focus first on the bond with your father’* or inviting the young person to decide what they preferred to talk about. Furthermore, peer counsellors provided brief summaries and clarified what they could and could not do for the young person.

Counselling techniques were often not used in isolation. Peer counsellors often first expressed their empathy and/or responded affirmingly, before asking a question or giving advice, for example: ‘*What an intense day indeed! What made your appointment so unpleasant?*’, ‘*I can imagine that's not nothing. Have you ever tried breathing exercises?*’ or ‘*It is okay to feel like this sometimes. What in life exactly are you not seeing [the value of] anymore?*’ and in the case of advice: ‘*My compliments for coming here on the chat with this! I think it should be a good idea for you to contact [organisation], have you heard about them yet?*’

In other instances, empathising/affirming was done exclusively (e.g. ‘*I can imagine that must have been so difficult for you’* or ‘*I mean, if you feel like you need to scream, scream. It's totally okay you're feeling this way right now’* or ‘*That must have been frightening! I think it is very brave that you are taking it all on in therapy!’*). Empathising/affirming could entail praising young people's openness (e.g. *‘You're doing a great job discussing this’*) or expressing confidence in their ability to manage challenges. In other moments, this could be an expression of a concern (e.g. *‘I'm genuinely concerned that you're experiencing vomiting blood; this is serious, and it's important to inform your doctor.’*). Empathising/affirming could be very brief, e.g. *‘That's wonderful!’* or *‘How delightful!’*, or more elaborate, e.g. *‘I'm hopeful that your talk with your mom will go well; you've got this!’* Moreover, empathising/affirming could be intended to normalize the young person's emotions concerning various topics, for example regarding intimacy or depression, that *‘It's completely normal to feel like that at times’*, or explicitly state their active listening and availability, e.g. *‘I'm all ear; feel free to talk about anything.’*

### 3.4 Responses by service users

Responses by young people who sought digital peer support varied from open-ended (i.e. elaborating, deepening the conversation) to closed-ended or evasive answers (i.e. brief dismissive or confirmatory replies or replies that evade the conversation), and from agreeing to disagreeing with suggestions or confirmatory questions by the peer counsellors. There was also a division between young people who logged on with a specific problem or question, and those who wanted to chat casually without a predetermined topic or expressed need. Various young people chose the direction of the online chat session, by their own initiative or when being invited to, or explained their thoughts of what could be helpful for them. Additional responses included the expression of gratitude, positive evaluation, asking for the opinion of the peer counsellor, or implementing advice (e.g. informing their mentor when struggling with a school-related matter) versus dismissing advice.

From the above-mentioned distinctions, instances where the young person disengaged were classified as low in collaboration (coded 0). This included: giving closed-ended or evasive answers, disagreeing without any further openness, expressing helplessness (i.e. a sense of inability to address challenges despite guidance), showing no interest in implementing advice, and expressing indifference about what the peer counsellors could do for them. Young people sometimes suddenly left the online chat, but the reasons for sudden departures were unknown and these were therefore not included as responses.

Conversely, instances where the young person responded with active participation and openness were classified as good in collaboration (coded 1). These responses included: opening up, elaborating on or deepening the topic, being in agreement with the counsellor, introducing new topics important to them, asking for information or guidance, implementing advice, expressing gratitude, explaining what had previously helped them or could help now, choosing the direction of the online chat session, evaluating the conversation positively, expressing feeling better or helped, asking for the view of the peer counsellors, expressing openness to visit @ease on-site, and expressing intent to return to the online chat in the future. Of the 623 responses provided by the young persons, 423 (67.9%) were collaborative responses.

### 3.5 Correlations

Correlations between counselling techniques all had a small magnitude, with the largest correlation between open-ended and closed-ended questions, which was negative and significant (*r* = −0.21, *p* < 0.001). Both types of questioning showed small significant correlations with multiple other counselling techniques (ranging up to *r* = − 0.20, *p* < 0.001). Giving advice was negatively, significantly, correlated with empathising/affirming alone (*r* = − 0.14, *p* < .001) and empathising/affirming combined with open-ended (*r* = − 0.09, *p* = 0.025) or closed-ended (*r* = − 0.11, *p* = 0.005) questions. Empathising/affirming further correlated negatively and significantly with the combinations of empathising/affirming and asking open-ended questions (*r* = − 0.12, *p* = 0.003), closed-ended questions (*r* = − 0.09, *p* = 0.022) and giving advice (*r* = − 0.10, *p* = 0.017), and with giving advice alone (*r* = − 0.14, *p* < 0.001).

Collaborative responding of the young person correlated significantly, positively, and to a small extent with the counsellors' techniques of empathising/affirming (*r* = 0.24, *p* < 0.001), asking an open-ended question (*r* = 0.10, *p* = 0.014), and the combination empathising/affirming with asking an open-ended question (*r* = 0.11, *p* = 0.005), while it correlated negatively with asking a closed-ended question (*r* = − 0.20, *p* < 0.001), the combination of empathising/affirming with asking a closed-ended question (*r* = − 0.12, *p* = 0.003) and with giving advice (*r* = − 0.13, *p* = 0.001), as well as with giving advice only (*r* = − 0.11, *p* = 0.008), as detailed in Supplementary Table 3.

### 3.6 Associations between peer-counselling techniques and type of response

The logistic regression analysis displayed a good fit of the model and a moderate pseudo-*R*^*2*^ of 21% (Hosmer-Lemeshow x^2^ = 0.47, *p* = 1.000, Nagelkerke *R*^*2*^ = 0.21). Empathising/affirming was positively associated with collaborative responses (*OR* = 4.13, 95%CI 1.53–11.16, *p* = 0.005). Conversely, collaborative responses were negatively associated with the counselling techniques: asking closed-ended questions (*OR* = 0.23, 95%CI 0.11–0.47, *p* < 0.001), the combination of empathising/affirming with asking closed-ended questions (*OR* = 0.25, 95%CI 0.12–0.54, *p* < 0.001), the combination of empathising/affirming with giving advice (*OR* = 0.17, 95%CI 0.07–0.46, *p* < 0.001), and giving advice only (*OR* = 0.27, 95%CI 0.12–0.61, *p* = 0.002), as detailed in Table 4.

**Table 4:** Associations between peer-counselling techniques and type of response in 631 exchanges.

	*OR*	*95% CI*		*P*
Asking an open-ended question	0.89	0.41	1.92	0.768
Asking a closed-ended question	0.23	0.11	0.47	<0.001
Checking understanding	0.46	0.15	1.39	0.167
Empathising/affirming	4.13	1.53	11.16	0.005
Empathising/affirming + asking an open-ended question	1.64	0.57	4.73	0.356
Empathising/affirming + asking a closed-ended question	0.25	0.12	0.54	<0.001
Empathising/affirming + giving advice	0.17	0.07	0.46	<0.001
Giving advice only	0.27	0.12	0.61	0.002
Structuring the conversation	0.50	0.19	1.32	0.163
*Constant*	4.05			<0.001

Abbreviations: OR = Odds Ratio (<1 negative; 1 > positive relationship), CI = Confidence Interval.

Note: Good collaboration was the reference category (coded 1); Bonferroni correction yielded a < 0.006; Solution-focused and self-disclosure/information techniques were not included due to low frequency of occurrence.

## 4 Discussion

This study explored the key topics, conversation techniques used by peer counsellors, and responses given by young people on the online chat of @ease, as well as the links between counselling techniques and responses. In this explorative study, empathising/affirming was positively associated with collaborative responses but empathising/affirming combined with advice-giving or asking a closed-ended question, or advice-giving only, was negatively associated with collaborative responses. While replication in a larger sample is necessary, current findings underline the importance of creating a sense of safety, understanding, encouragement, hope, and comfort, which empathising/affirming consisted of, without following up directly with a question or advice.

The key topics in the @ease online chat sessions paralleled the on-site topics brought forward during walk-in visits, supporting the first hypothesis. Frequently discussed topics included social and occupational problems and problems related to parents, which may be explained by the changes that occur in this developmental period, including increasing academic responsibilities and redefined relationships with parent(s)/caregiver(s) and peers (Tetzner et al., 2017). Generally, performance at school has been a core theme of worry for young people across cultures (Owczarek et al., 2020). Furthermore, compared to walk-in visits, the topic of intimacy and sexuality more frequently appears to be a main reason for presenting on the online chat. Sexual exploration, and related aspects such as the start of contraceptive use, often start in this age range (Gilmore and Meersand, 2023; Lindberg et al., 2021) and discussing this in an online chat environment may feel more anonymous and safer.

Moreover, professional help and diagnoses, and suicidality, were two main topics that young people accessed online peer counselling for. Both topics are highly applicable to this age range, as mental disorders form primarily before the age of 25 years (Solmi et al., 2022), and because suicide is the most prevalent cause of death among young people (Statistics Netherlands, 2023). In 2023, 14% of young people aged 12–25 years were reported to have suicidal thoughts in the Netherlands, especially among those experiencing emotional loneliness (National Institute for Public Health and the Environment [RIVM], 2023). Among visitors of the @ease walk-in centres from 2018 to 2022, almost 30% experienced suicidal ideations (Boonstra et al., 2024b), suggesting that young people who accessed peer counselling at @ease may have been struggling with suicidal thoughts with greater likelihood than youth in the general population. Collectively, these expressed concerns underscore the importance of an accessible (online) space where young people can talk openly about these topics. Additionally, peer-to-peer interventions may aid in reducing stigma (Sun et al., 2022).

In response to the concerns voiced by online chat visitors, the peer counsellors applied various techniques to guide active listening, such as asking questions, empathising/affirming, and providing self-disclosure. Empathising/affirming was the only factor that was positively linked with collaborative responses, and this technique encompassed, for example, providing emotional support, acknowledging feelings, showing genuine concern, encouraging, instilling hope, and normalizing, without judgment. While replication is needed to draw conclusions, these explorative findings align with literature and practice. The importance of empathic responding in therapy has long-since been described, in which empathy is considered as fundamental for therapeutic change, putting empathy at the centre of psychotherapy, and of development, as caregivers' empathy has been considered vital for wellbeing from birth (e.g. Kohut, 1977; Rogers, 1957). Moreover, empathic responding, as core aspect of active listening (Nemec et al., 2017), showed a moderate association with therapeutic alliance across 53 studies (Nienhuis et al., 2018) and a moderately strong association with psychotherapy outcomes across 82 samples (Elliott et al., 2018). It must be noted that most studies focused on *perceived* empathy by the care receiver rather than externally coded empathy, yet results seem to align.

In digital settings, fewer studies exist that assess the impact of empathising and/or affirming. An example stems from internet-based cognitive behaviour therapy (ICBT), where affirming, defined as validating, normalizing, and summarizing, as well as encouraging, were found to be significantly correlated with improvements in depressive symptoms (Holländare et al., 2016). Several other studies on the topic of online counselling and empathy were focused on chatbots. Emotional awareness and empathising have interestingly been raised as key concerns for shortcomings of artificial intelligence (AI) chatbots (Pham et al., 2022). Yet, newer studies are finding that AI chatbots are perceived as more empathetic than human healthcare specialists (Howcroft et al., 2025). On the other hand, perceived inauthenticity of the chatbots seems to hamper trust in them (Seitz, 2024), and, relatedly, a chatbot cannot offer the benefit of speaking with a real peer that has been through resembling experiences. Moreover, Sharma et al. (2023) researched the use of AI by peer counsellors and found that AI feedback increased the expressed empathy by peer counsellors, even more so among peer counsellors who voiced difficulties in providing support, thereby proposing AI as a tool rather than a replacement.

Advice-giving yielded poor collaborative responses, also when combined with empathising/affirming. Earlier research concordantly found that active listening tends to make people feel more understood than when receiving advice (Weger Jr et al., 2014). Giving advice has also been linked with poor subsequent client collaboration in psychotherapy, meaning that the extent to which clients could address issues and use interventions by means of this technique was low (Prass et al., 2021). Furthermore, as expected, closed-ended questions were associated with poor collaborative responses, even when combined with empathising/affirming. When confronted with a question that only required a brief answer, usually only a brief answer was given, and young people seemed to not take the space to elaborate. Asking open-ended questions was surprisingly not associated with response type, also when combined with empathising/affirming, meaning that the given space to elaborate was likely sometimes taken and sometimes dismissed. Elaboration was much more likely to follow after empathising/affirming without any additions; a purely welcoming and understanding atmosphere helped to open up.

Other factors were not associated or were applied too infrequently to analyse reliably. For absent associations, it must be noted that young people's responses were coded specifically to learn about whether and which peer support interventions might deepen a conversation in online chat counselling. However, some interventions might not immediately facilitate more openness or depth but may contribute to other valuable aspects of peer counselling. Hence, other counselling techniques should not be regarded as inefficient overall, but rather inefficient in deepening the conversation according to the present subsample. For example, while checking understanding and structuring the conversation were not significantly associated with response type, these can still be helpful conversation techniques for the counsellors to understand and thereby support the young person better and improve the clarity of the conversation, without the effect being noticeable in the immediate response given by the service user. Next, solution-focused and self-disclosure/information-giving techniques were only used 16 and 12 times, respectively, out of 595 counselling responses throughout this subset of online chat sessions. This is surprising, given that both elements are important parts of the training and working method. It might be that the peer counsellors find these techniques more difficult in the online setting and may value more attention to this in the trainings and guidance by the on-site professionals. Researching the experiences of peer counsellors regarding the online chat could help clarify challenges and improve practice further.

## 5 Strengths and limitations

In this digital age, and especially for young people, peer support chat services are both timely and relevant. The present explorative study provided a first overview of topics, techniques, and responses arising in a peer support chat service for young people aged 12 to 25 years, without a boundary at the age of 18 years. While digital approaches are highly accessible, especially for young people, studies on online peer support services are scarce. The results of the present study add to current knowledge for the training of peer counsellors to best support young people with mental health problems and inform future analyses.

The methodology of this study has both strengths and limitations. Latent content analysis facilitates the investigation of textual information (Graneheim et al., 2017), and can thus be highly ecological, providing insight into naturally occurring processes and reducing chances of desirable responding in case of a random subsample. Through reported quotations linked to the main counselling techniques and their context, major themes could be presented thoroughly and reported findings showed consistency with the data. Furthermore, a core characteristic of qualitative methods is that the coder plays an active role in assigning and combining codes, influencing the identification and interpretation of outcomes. While necessary, this active role can have drawbacks such as coder fatigue or reduced ease of understanding of codes to the readers (Kleinheksel et al., 2020). Furthermore, comparability of the data is limited, especially with walk-in visits, as in-person counselling sessions were not transcribed. Only the main topics can be compared narratively.

Another limitation is found in the number of online chat sessions included in this study, which necessitates caution in drawing firm conclusions. This study is exploratory and intended to inform large-scale analyses. In addition, the eventual frequencies did not allow for topic-specific analyses, and not all subtopics were observed which might have been expected based on previous reporting on young people's concerns. While all anonymous online chat sessions could be coded to derive frequencies of occurrence, this undertaking would be large if coding manually. Current advancements such as large language models pose options for more extensive future analyses, possibly increasing generalizability and dependability on implications (Abdurahman et al., 2024).

The reached age groups by the @ease online chat were more balanced than the age groups accessing the @ease walk-in centres in the first operational years. Specifically, between 2018 and 2022, most of the walk-in visitors were aged 18 to 25 years (72% of one-time visitors and 56% of returning visitors) (Boonstra et al., 2024b), while 40% were aged 18 to 25 years in the presently analysed online chat data of 2023. For walk-in visits, the overrepresentation of older young people happened due to having started up in large university cities and more balance is expected through efforts over time. However, not all young people who need peer support may access the @ease walk-in centres or online chat. Some young persons may not have access to transport, internet, or a device, may be unaware of @ease, or experience barriers to find or access support by themselves. Moreover, young people who identify as male or non-binary remain underrepresented. To reach a more diverse population, @ease also employed a community youth mental health outreach program, termed Everybody @ease (Crombach et al., 2025).

## 6 Implications and future research

The finding that empathic and affirming responses outperformed advice-giving or other additions aligns well with literature and can thereby be used for improving training and guidance of the peer counsellors by the on-site professional. If replicated, this research can offer actionable steps to better prepare peer counsellors to provide meaningful support in an online environment. These initial findings allow for informed research of intervention effectiveness in the future. For future research, the use of large language models may allow for a far larger dataset to be analysed, and split per topic, provided it is achieved anonymously and securely.

When analysing a large dataset of online chat sessions in the future, clarity can be gained on the main topics and on whether techniques are used infrequently at the online chat overall, including whether they could have been used more often, and what their influence might be on young people's responses. While the @ease training is already highly focused on these counselling techniques (e.g. solution-focused techniques, providing self-disclosure), both the trainings as well as the guidance by the on-site professional may need to involve discussion and practice of what this might look like in an online setting, as well as when it is suitable and appropriate, and encourage it further in those instances.

## 7 Conclusions

This explorative study provided first insights into the arising topics, applied peer counselling techniques, and responses by young people accessing an online chat service for peer support. When peer counsellors actively listened through empathising/affirming, without directly asking a question or offering advice, this helped young people to open up when accessing online chat peer counselling. By understanding the frequency and impact of counselling techniques, mental health support services can better train counsellors and tailor their approaches to meet the needs of young people, ultimately enhancing the support provided through online platforms such as @ease.

## Funding

@ease has received funding from the participating municipalities, health care partners (in kind), the Dutch Health Insurers Innovation Fund (Innovatiefonds Zorgverzekeraars), 10.13039/501100001826ZonMW and the 10.13039/501100002999Dutch Ministry of Health, Welfare and Sport (dedicated to SL, number 06360332210024, Ministerie van Volksgezondheid, Welzijn en Sport).

## Declaration of competing interest

The authors declare the following financial interests/personal relationships which may be considered as potential competing interests: The authors affiliated with the @ease consortium and MHeNs were active in the @ease Foundation as professionals and/or advisory board members. There are no further interests to declare.

@ease has received funding from the participating municipalities, health care partners (in kind), the Dutch Health Insurers Innovation Fund (Innovatiefonds Zorgverzekeraars), ZonMW and the Dutch Ministry of Health, Welfare and Sport (dedicated to SL, number 06360332210024, Ministerie van Volksgezondheid, Welzijn en Sport). If there are other authors, they declare that they have no known competing financial interests or personal relationships that could have appeared to influence the work reported in this paper.

## Data Availability

Authors elect to share no qualitative data due to the privacy and ethical constraints of the anonymous online chat sessions while quantitative data are available from the corresponding author upon reasonable request.
