# Supplementary material for: Youth supporting youth online: A content analysis of peer counselling at the online chat of @ease
Source: Internet Interv. 2026 Apr 1;44:100940. doi: 10.1016/j.invent.2026.100940 (PMC13087644; doi:10.1016/j.invent.2026.100940)
Supplement: Supplementary file 1 — Supplementary tables [file mmc1.pdf]

Supplementary Table 1. *COREQ (COnsolidated criteria for REporting Qualitative research) Checklist*

Topic	Item No.	Guide Questions/Description	Reported on Page No.
**Domain 1: Research team and reflexivity**			
Personal characteristics			
Interviewer/facilitator	1	Which author/s conducted the interview or focus group?	Analyses, p. 6
Credentials	2	What were the researcher's credentials? E.g. PhD, MD	Analyses, p. 6
Occupation	3	What was their occupation at the time of the study?	Analyses, p. 6
Gender	4	Was the researcher male or female?	Analyses, p. 6
Experience and training	5	What experience or training did the researcher have?	Analyses, p. 6
Relationship with participants			
Relationship established	6	Was a relationship established prior to study commencement?	Analyses, p. 6
Participant knowledge of the interviewer	7	What did the participants know about the researcher? e.g. personal goals, reasons for doing the research	N/A (No interviews)
Interviewer characteristics	8	What characteristics were reported about the inter viewer/facilitator? e.g. Bias, assumptions, reasons and interests in the research topic	N/A (No interviews)
**Domain 2: Study design**			
Theoretical framework			
Methodological orientation and Theory	9	What methodological orientation was stated to underpin the study? e.g. grounded theory, discourse analysis, ethnography, phenomenology, content analysis	Analyses, p. 5 (Content analysis)
Participant selection			
Sampling	10	How were participants selected? e.g. purposive, convenience, consecutive, snowball	Analyses, p. 6 (consecutive)
Method of approach	11	How were participants approached? e.g. face-to-face, telephone, mail, email	N/A (accessed us)
Sample size	12	How many participants were in the study?	Results, p. 6
Non-participation	13	How many people refused to participate or dropped out? Reasons?	N/A
Setting			
Setting of data collection	14	Where was the data collected? e.g. home, clinic, workplace	N/A (digital)
Presence of nonparticipants	15	Was anyone else present besides the participants and researchers?	Procedure, p. 4
Description of sample	16	What are the important characteristics of the sample? e.g. demographic data, date	Table 1, p. 5 + Characteristics, p. 6
Data collection			
Interview guide	17	Were questions, prompts, guides provided by the authors? Was it pilot tested?	N/A (No interviews)
Repeat interviews	18	Were repeat interviews carried out? If yes, how many?	N/A (No interviews)
Audio/visual recording	19	Did the research use audio or visual recording to collect the data?	N/A (No interviews)

Field notes	20	Were field notes made during and/or after the interview or focus group?	N/A (No interviews)
Duration	21	What was the duration of the interviews or focus group?	Characteristics, p. 6
Data saturation	22	Was data saturation discussed?	Analyses, p. 6
Transcripts returned	23	Were transcripts returned to participants for comment and/or correction?	N/A (Anon)
**Domain 3: analysis and findings**			
Data analysis			
Number of data coders	24	How many data coders coded the data?	Analyses p. 6
Description of the coding tree	25	Did authors provide a description of the coding tree?	Supplementary Table 2, p. 17-18
Derivation of themes	26	Were themes identified in advance or derived from the data?	Analyses p. 5-6 (Hybrid)
Software	27	What software, if applicable, was used to manage the data?	Analyses p. 5-6 (Atlas.ti)
Participant checking	28	Did participants provide feedback on the findings?	N/A (Anon)
Reporting			
Quotations presented	29	Were participant quotations presented to illustrate the themes/findings?<br><br>Was each quotation identified? e.g. participant number	Results, p. 7-8 + Supplementary File 2<br><br>No, anon
Data and findings consistent	30	Was there consistency between the data presented and the findings?	Results, p. 7-8 + Supplementary File 2, Strengths and limitations, p. 11
Clarity of major themes	31	Were major themes clearly presented in the findings?	Results, p. 7-8 + Supplementary File 2, Strengths and limitations, p. 11
Clarity of minor themes	32	Is there a description of diverse cases or discussion of minor themes?	Discussion, p. 11

Developed from: Tong A, Sainsbury P, Craig J. Consolidated criteria for reporting qualitative research (COREQ): a 32-item checklist for interviews and focus groups. *International Journal for Quality in Health Care*. 2007. Volume 19, Number 6: pp. 349 – 357.

Supplementary Table 2. *Categories and definitions of peer counselling techniques*

Counselling technique categories	Definition
Asking questions	
Asking open-ended questions	Asking a question that allows for detailed and open-ended responses, encouraging the young person to express themselves fully.<br>- For example: “*How are you feeling right now?”*
Asking closed-ended questions	Asking a question that typically demands a short or single-word answer. This category also includes semi-closed questions that provide guidance toward the expected reply and/or encourage elaboration.<br>- For example: “*Did you enjoy the experience?”*
Checking understanding	Confirming one’s comprehension or confirming details, including verifying information or ensuring that the young person's statements were accurately understood by the counsellor.<br>- For example: “*So you went to the pool with your class, is that correct?”*
Empathising/affirming	
Empathising/affirming only	Demonstrating understanding and emotional support while fostering a sense of comfort by acknowledging the young person's feelings, showing genuine concern, agreeing, offering encouragement, expressing concern without judgement, providing positive affirmations, instilling hope, normalizing feelings, offering a listening ear, or paraphrasing words as affirmation.<br>- For example: “*I mean, if you feel like you need to scream, scream. It's totally okay you're feeling this way right now.”* or “*I can imagine that must have been so difficult for you.”* or “*That must have been frightening! I think it is very brave that you are taking it all on in therapy!”*
Empathising/affirming and asking a question	Empathising/affirming while also asking questions to gain further insight or gain clarification of the young person’s situation.<br>- For example: “*What an intense day indeed! What made your appointment so unpleasant?” or “It is okay to feel like this sometimes. What in life exactly are you not seeing anymore?”*
Empathising/affirming and giving advice	Empathising/affirming while also providing advice and suggesting possible actions or solutions.<br>For example: “*I can imagine that's not nothing. Have you ever tried breathing exercises?”* or “*My compliments for coming here on the chat with this! I think it should be a good idea for you to contact ‘Veilig Thuis’, have you heard from them yet?”*
Giving advice	Counselling techniques where peer counsellors offer specific advice or encourage the young person to do something.<br>For example: “*I would really advise you to discuss this problem with your mentor as well.”*

Structuring the conversation	Clarifying how peer counsellors can help, setting boundaries regarding what they are (not) able and/or allowed to do in their position, inviting the young person to guide the conversation, and organizing the dialogue to make it more coherent and effective.<br>For example: “*You name several things. I would like to focus first on the bond with your father.”*
Providing self-disclosure/information	Instances where peer counsellors share their own experiences or provide information resembling psychoeducation to support the young person. ‘Information’ here was based on the peer counsellors' personal knowledge and experience, such as explanations of the characteristics of menstruation.<br>For example: “*Menstrual cramps often come to me in waves.”*
Stimulating solution-focused thinking	Encouraging the young person to think of solutions, for example by reflecting on what has previously helped them and considering new solutions.<br>For example: “*Do you think you could do something that would make you less nervous?”*

Supplementary Table 3. *Correlations between all counselling techniques and between counselling techniques and collaborative response within the online chat sessions (n=26).*

	1	2	3	4	5	6	7	8	9	10	11	12
1. Asking an open-ended question	1.0											
2. Asking a closed-ended question	-.21**	1.0										
3. Checking understanding	-.08*	-.08*	1.0									
4. **E**mpathising/affirming	-.20**	-.20**	-.08	1.0								
5. **E**mpathising/affirming + asking a closed-ended question	-.16**	-.16**	-.06	-.09*	1.0							
6. **Empathising/affirming** + asking an open-ended question	-.13**	-.13**	-.05	-.12**	-.10*	1.0						
7. **E**mpathising/affirming + giving advice	-.10**	-.10**	-.04	-.10*	-.08	-.06	1.0					
8. Giving advice only	-.15**	-.15**	-.06	-.14**	-.11**	-.09*	-.07	1.0				
9. Stimulating solution-focused thinking	-.07	-.08	-.03	-.07	-.06	-.05	-.04	-.05	1.0			
10. Structuring the conversation	-.10**	-.11**	-.04	-.08	-.08*	-.06	-.05	-.07	-.04	1.0		
11. Providing self-disclosure/information	-.06	-.07	-.03	-.06	-.05	-.04	-.03	-.04	-.02	-.03	1.0	
12. Collaborative response	.10*	-.20**	-.01	.24**	-.12**	.11**	-.13**	-.11**	.02	.00	.07	1.0

Notes: * *p ≤ .05*, ** *p ≤ .01*
